# Supplementary material for: Patterns of Intron Gain and Loss in Fungi
Source: PLoS Biol. 2004 Nov 30;2(12):e422. doi: 10.1371/journal.pbio.0020422 (PMC532390; doi:10.1371/journal.pbio.0020422)
Supplement: Table S1 — Also available at http://genes.mit.edu/NielsenEtAl/. (4.3 MB ZIP). [file pbio.0020422.st001.zip › NielsenEtAl/html/782.html]

AN5129.1.NCU09602.1.MG06958.2.FG00838.1


```
 CLUSTAL W (1.82) Multiple Sequence Alignments - Introns Inserted


Sequence 1: NCU09602.1	646 aa
Sequence 2: MG06958.2	651 aa
Sequence 3: FG00838.1	653 aa
Sequence 4: AN5129.1	644 aa
Alignment Length: 654 aa
Number Identitical Residues: 507 aa
Alignment Score (without introns) 20433


MG06958.2 	MAPAVGIDLGTTYSCVGIFRDDR~IE0IIANDQGNRTTPSFVAFTDTERLIGDAAKNQVA
NCU09602.1	MAPAVGIDLGTTYSCVGVFREDR2CE~IIANDQGNRTTPSFVAFTDTERLVGDAAKNQVA
FG00838.1 	MGPAVGIDLGTTYSCVGIFREDR2CD~IIANDQGNRTTPSFVGFTDTERLIGDAAKNQVA
AN5129.1  	MAPAVGIDLGTTYSCVGVFRDDR~ID~IIANDQGNRTTPSFVAFTDTERLIGDAAKNQVA
          	*.***************:**:**  : ***************.*******:*********

MG06958.2 	MNPHNTVFDAKRLIGRKFSDAEVQADMKHFPFKVVERSGKPVIEVEFKGESKQFTPEEIS
NCU09602.1	MNPANTVFDAKRLIGRKFSDPEVQADMKHFPFKVIDRGGKPVIQVEFKGETKVFTPEEIS
FG00838.1 	MNPQNTVFDAKRLIGRKFADAEVQADMKHFPFTIIDKAGKPAIEVEFKGEKKTFTPEEIS
AN5129.1  	MNPHNTVFDAKRLIGRRFGDAEVQADMKHWPFKVVDKSGKPIIEVEFKGETKQFTPEEIS
          	*** ************:*.*.********:**.::::.*** *:******.* *******

MG06958.2 	SMVLTKMRETAESYLGGTVNNAVVTVPAYFNDSQRQATKDAGLIAGLNVLRIINEPTAAA
NCU09602.1	AMILQKMKETAEAYLGGTVNNAVVTVPAYFNDSQRQATKDAGLIAGLNVLRIINEPTAAA
FG00838.1 	AMILTKMRETAESYLGETVNNAVVTVPAYFNDSQRQATKDAGLIAGLNVLRIINEPTAAA
AN5129.1  	SMVLTKMRETAEAFLGGTVNNAVITVPAYFNDSQRQATKDAGLIAGLNVLRIINEPTAAA
          	:*:* **:****::** ******:************************************

MG06958.2 	IAYGLDKKVEGERNVLIFDLGGGTFDVSLLTIEEGIFEVKSTAGDTHLGGEDFDNRLVTH
NCU09602.1	IAYGLDKKVEGERNVLIFDLGGGTFDVSLLTIEEGIFEVKSTAGDTHLGGEDFDNRLVNH
FG00838.1 	IAYGLDKKVEGERNVLIFDLGGGTFDVSLLTIEEGIFEVKSTAGDTHLGGEDFDNRLVNH
AN5129.1  	IAYGLDKKVEGERNVLIFDLGGGTFDVSLLTIEEGIFEVKATAGDTHLGGEDFDNRLVNH
          	****************************************:*****************.*

MG06958.2 	FANEFKRK~HKK0DLTTNARALRRLRTACERAKRTLSSSAQTSIEIDSLYEGIDFYTSIT
NCU09602.1	FVQEFKRK0HKK~DLSTNARALRRLRTACERAKRTLSSSAQTSIEIDSLFEGIDFYTSIT
FG00838.1 	FVNEFKRK~HKK~DLSTNVRALRRLRTACERAKRTLSSSAQTSIEIDSLFEGIDFYTSIT
AN5129.1  	FVTEFKRK~HKK~DLSTNARALRRLRTACERAKRTLSSAAQTSIEIDSLFEGIDFYTSIT
          	*. ***** *** **:**.*******************:**********:**********

MG06958.2 	RARFEELCQDLFRSTLQPVDRVLTDAKIDKAQVHEIVLVGGSTRIPRIQKLITDYF~NGK
NCU09602.1	RARFEELCQDLFRSTLQPVDRVLTDAKIDKSQVHEIVLVGGSTRIPRIQKLISDYF0NGK
FG00838.1 	RARFEELCQDLFRSTIQPVDRVLTDAKIDKSLVHEIVLVGGSTRIPRVQKLITDYF~NGK
AN5129.1  	RARFEELCQDLFRGTMEPVERVLRDAKIDKSSVHEIVLVGGSTRIPKIQRLVSDYF~N-K
          	*************.*::**:*** ******: **************::*:*::*** * *

MG06958.2 	EPNKS~INPDEAVAYGAAVQAAILSGDTSSKSTNEILLLDVAPLSLGIETAGGMMTKLIP
NCU09602.1	EPNKS2INPDEAVAYGAAVQAAILSGDTSSKSTSEILLLDVAPLSLGIETAGGMMTKLIP
FG00838.1 	EPNKS~INPDEAVAYGAAVQAAILSGDTSSKATNEILLLDVAPLSLGIETAGGMMTKLIP
AN5129.1  	EANKS~INPDEAVAYGAAVQAAILSGDTSSKSTNEILLLDVAPLSVGIETAGGVMTPLVK
          	*.*** *************************:*.***********:*******:** *: 

MG06958.2 	RNTTIPTKKSEVFSTFSDNQPGVLIQVYEGERQRTKDNNLLGKFELTGIPPAPRGVPQIE
NCU09602.1	RNTTIPTKKSEVFSTFSDNQPGVLIQVYEGERQRTKDNNLLGKFELTGIPPAPRGVPQIE
FG00838.1 	RNTTIPTKKSEVFSTFSDNQPGVLIQVYEGERQRTKDNNLMGKFELTGIPPAPRGVPQIE
AN5129.1  	RNTTIPTKKSETFSTYSDNQPGVLIQVYEGERARTKDNNLLGKFELTGIPPAPRGVPQIE
          	***********.***:**************** *******:*******************

MG06958.2 	VTFDLDANGIMNVSAVEKGTGKTNQITITNDKGRLSKEDIERMLAEAEKFKEEDEAEAAR
NCU09602.1	VTFDVDANGIMNVSALEKGTGKTNQITITNDKGRLSKEEIERMLAEAEKFKEEDEAEAKR
FG00838.1 	VTFDLDANGIMNVSAVEKGTGKSNKIVITNDKGRLSKEEIERMLNDAEKYKEEDEAEGKR
AN5129.1  	VTFDLDANGIMNVSAVEKGTGKTNKITITNDKGRLSKEDIERMLADAEKYKAEDEAEAAR
          	****:**********:******:*:*.***********:***** :***:* *****. *

MG06958.2 	VSAKNGLESYAYSLRNTLQDSKVDEKLDAADKEKLNAEINKIVSWLDESQQATKEEYEEH
NCU09602.1	VAAKNGLESYAYSLRNTLSDSKVDEKLDAADKEKLKSEIDKIVAWLDENQQATREEYEER
FG00838.1 	VAAKNGLESYAYSLRNTLSDPKVEEKIEASDKETLTAEIDKVVQWLDDNQQATREEYEEH
AN5129.1  	IQAKNGLESYAYSLKNTISEG--QLQISEDDKKKVSDKIDEVISWLDNNQTAEKDEYESQ
          	: ************:**:.:   : ::.  **:.:. :*:::: ***:.* * ::***.:

MG06958.2 	QKELEAVANPIMMKFYGAGG--APGGMPGAPGGAPGGFPGA-GGAPGAGGDDGPTVEEVD
NCU09602.1	QKELEAIANPIMMKFYGAGG--APGGMP---GAAPGGFP---GGAPGSNDNEGPTVEEVD
FG00838.1 	QKELEGKANPIMMKFYGAGGEGAPGGMPG-MPGGPGGFPGAGGPAPGAGGDDGPTVEEVD
AN5129.1  	QKELEGVANPIISAAYAAAG-GAPGGAAPGAGAAPG------GGAGFRNDGVVEENEELD
          	*****. ****:   *.*.* .**** . .  ..**      * *   ...     **:*

NCU09602.1	
MG06958.2 	
FG00838.1 	
AN5129.1  	
          	
```
